# Deciphering SARS-CoV-2 Virologic and Immunologic Features

**DOI:** 10.3390/ijms21165932

**Published:** 2020-08-18

**Authors:** Grégorie Lebeau, Damien Vagner, Étienne Frumence, Franck Ah-Pine, Xavier Guillot, Estelle Nobécourt, Loïc Raffray, Philippe Gasque

**Affiliations:** 1 Unité de Recherche Études Pharmaco-Immunologiques, Centre Hospitalier Universitaire La Réunion Site Félix Guyon, CS11021, 97400 Saint Denis de La Réunion, France; damien.vag@gmail.com (D.V.); etienne.frum@gmail.com (É.F.); xavier.guillot@chu-reunion.fr (X.G.); Philippe.gasque@chu-reunion.fr (P.G.); 2 Laboratoire de Biologie, Secteur Laboratoire d’immunologie Clinique et Expérimentale de la Zone de l’océan Indien (LICE-OI), Centre Hospitalier Universitaire La Réunion Site Félix Guyon, CS11021, 97400 Saint Denis de La Réunion, France; 3 Unité Mixte de Recherche Processus Infectieux en Milieu Insulaire Tropical (PIMIT), Université de La Réunion, INSERM UMR 1187, CNRS 9192, IRD 249, Platform CYROI, 2 rue Maxime Rivière, 97491 Sainte Clotilde, La Réunion, France; 4 Service d’anatomo-Pathologie, Centre Hospitalier Universitaire Sud Réunion, 97410 Saint Pierre, France; franck.ahpine@gmail.com; 5 Service de Rhumatologie, Centre Hospitalier Universitaire La Réunion Site Félix Guyon, CS11021, 97400 Saint Denis de La Réunion, France; 6 Service d’endocrinologie Diabétologie, Centre Hospitalier Universitaire Sud Réunion, 97410 Saint Pierre, France; estelle.nobecourt@chu-reunion.fr; 7 Université de Formation et de Recherche Santé, Université de la Réunion, 97400 Saint-Denis, France; 8 Service de Médecine Interne, Centre Hospitalier Universitaire La Réunion Site Félix Guyon, CS11021, 97400 Saint Denis de La Réunion, France; loic.raffray@chu-reunion.fr

**Keywords:** SARS-CoV-2, COVID-19, virus biology, immunopathology, immunotherapy

## Abstract

Severe acute respiratory syndrome coronavirus (SARS-CoV)-2 and its associated pathology, COVID-19, have been of particular concerns these last months due to the worldwide burden they represent. The number of cases requiring intensive care being the critical point in this epidemic, a better understanding of the pathophysiology leading to these severe cases is urgently needed. Tissue lesions can be caused by the pathogen or can be driven by an overwhelmed immune response. Focusing on SARS-CoV-2, we and others have observed that this virus can trigger indeed an immune response that can be dysregulated in severe patients and leading to further injury to multiple organs. The purpose of the review is to bring to light the current knowledge about SARS-CoV-2 virologic and immunologic features. Thus, we address virus biology, life cycle, tropism for many organs and how ultimately it will affect several host biological and physiological functions, notably the immune response. Given that therapeutic avenues are now highly warranted, we also discuss the immunotherapies available to manage the infection and the clinical outcomes.

## 1. Introduction

A series of coronaviruses crossed the species barrier, with the severe acute respiratory syndrome coronavirus (SARS-CoV) in 2003 and the Middle East respiratory syndrome coronavirus (MERS-CoV) in 2012, and they caused major outbreaks of lethal pneumonia. Now, a new coronavirus emerged in December 2019 in Hubei province and was named the severe acute respiratory syndrome coronavirus 2 (SARS-CoV-2) virus. The disease caused by this virus, called COVID-19 has spread worldwide causing as early as August 2020 more than 18 million cases and more than 700,000 deaths [1].

SARS-CoV-2 is an enveloped virus with a positive RNA genome belonging to the beta genus of the Coronaviridae family [2].

The clinical features of COVID-19 range from asymptomatic or mild disease to critically life-threatening ill patients conditions. The most prevalent symptoms are fever, shortness of breath, dyspnea, cough and fatigue [3] and are commonly associated with diarrhea, headache, anosmia, lymphopenia or liver injury. An increasing number of less common manifestations are being described like acute kidney injury, vasculitis or myocarditis [4]. The most common comorbidities in critically ill patients are age, hypertension, diabetes, obesity, cardiovascular disease and respiratory system disease [3].

Viral and host interaction plays a key role in the pathogenesis of the disease. With the pandemic diffusion, better knowledge of the viral life cycle, cell tropism and host immune response is needed to find treatment against the disease. Here, we review the current understanding of the viral cycle, immunopathology as well as the current progress of immunotherapy.

## 2. Viral Description

### 2.1. Unravelling SARS-CoV-2 Genome and Viral Protein Features

SARS-CoV-2 (previously named new coronavirus 2019, nCoV-2019) is a single-stranded positive RNA virus, classified among the family Coronaviridae, within the Nidovirales order [2]. More precisely, SARS-CoV-2 belongs to the subgenus Sarbecovirus, within the *Betacoronavirus* genera and the subfamily Orthocoronavirinae [5,6]. Like other coronaviruses, SARS-CoV-2 was shown to possess a long genome, near to 30 kb, which showed 80% similarity with SARS-CoV, 50% with MERS-CoV and more identity with bat related coronaviruses (88% with bat-SL-CoVZC45 and bat-SL-CoVZXC21, 93% with RaTGT13) [6,7,8,9]. The first 2/3 parts of the genome encode the replicase–transcriptase complex, and the last third of the genome the four structural proteins [5,8,9]. Among all viral proteins, the replicase–transcriptase complex is the only one directly translated from the genome. ORF1a and ORF1b encode for two polyproteins (pp1a and pp1b) autoproteolytically processed to give the 16 non-structural proteins [5,10]. Whereas the structural proteins spike (S), envelope (E), membrane (M), nucleocapsid (N) and other accessory ones are expressed from subgenomic mRNAs [5,10].

The SARS-CoV-2 virion has a diameter of approximately 125 nm. The virion surface mainly bears the M protein and is poor in the E protein, however this last one is crucial for intracellular membrane assembly [5,11,12,13]. Moreover, the virion surface presents the spike protein (S), a highly glycosylated petal-shaped protein, composed of two functional domains S1 and S2 responsible for binding to the host cell receptor and membrane fusion respectively. The long genome is associated with helically symmetric N protein to form a nucleoprotein (NP), enclosed in the virion envelope. As SARS-CoV-2 has been recently identified, little is known about a large part of its proteins (structural and non-structural). However, growing efforts are made for this purpose. The SARS-CoV-2 spike protein is one of the most described, as it is the one that gives its infectivity to the virus. Spikeless coronaviruses exert poor infectivity [5]. Hence, this protein is essential for viral entry, as it binds to Angiotensin Converting Enzyme II—ACE2 as an entry receptor on the host cell, through its receptor-binding domain (RBD) located in the S1 subunit [14]. This system of entry is shared with SARS-CoV [15,16,17,18]. Additionally, this S protein is one of the major antigens [5], against whom is directed the neutralizing antibodies [14,19]. Notably a highly conserved epitope has been identified [20], which may explain cross-neutralization by SARS-CoV S-specific antibodies [14,17,19].

Of note, near-atomic structure of SARS-CoV-2 nsp12-nsp7-nsp8 complex (respectively catalytic subunit and two cofactors), representing the core polymerase complex, has been described and highly resembles SARS-CoV. Additionally, this gives insights about a better adaptation to the human host than SARS-CoV polymerase [21], maybe explaining the increased ribonucleic acid (RNA) production compared to SARS-CoV [22]. Finally, the SARS-CoV-2 main protease (Mpro) X-ray structure has also been reported [23]. Among the viral proteins that we presented, some are potential targets for novel drug development or drug repurposing, with already established lead compounds, notably due to computational methods [18,23,24]. The antiviral activity and clinical benefits of these compounds need to be investigated.

### 2.2. Inside the SARS-CoV-2V Life Cycle

#### 2.2.1. Entry and Fusion

**Figure 1:**
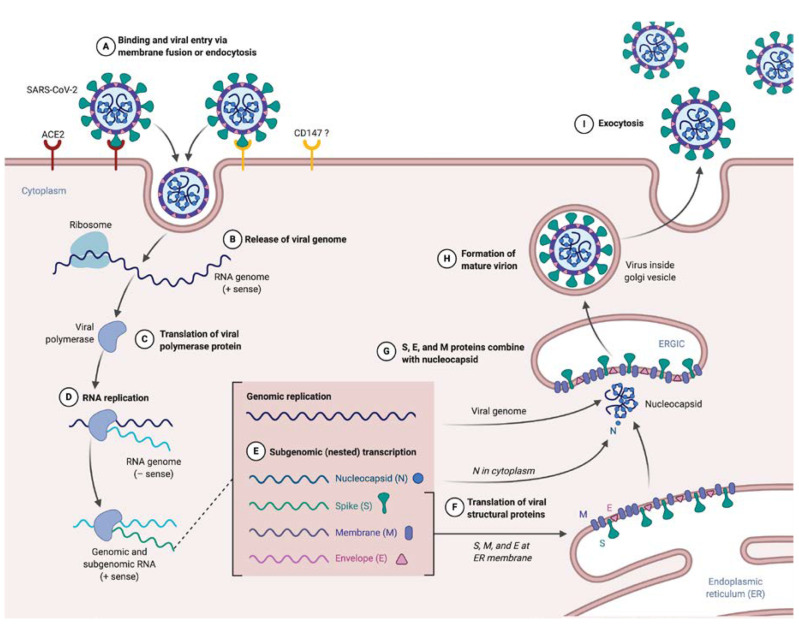
SARS-CoV-2 supposed life cycle. (**A**) Entry of SARS-CoV-2 in target cell expressing ACE2 (or another receptor, CD147 have been evoked but need to be confirmed). (**B**) Uncoating and releasing SARS-CoV-2 single stranded positive RNA genome. (**C**) Translation of replicase–transcriptase complex directly from RNA genome. (**D**) RNA genome replication due to a negative template. (**E**) Nested production of subgenomic RNA encoding for structural proteins. (**F**) Translation of viral S, E and M inserted in endoplasmic reticulum. (**G**) Nucleocapsid coupled to the genome, forming nucleoprotein, combine to S, E and M to form a mature virion (**H**). (**I**) Exocytosis of SARS-CoV-2.

SARS-CoV-2 is an airborne virus, meaning that it can be transmitted through droplets or contacts. Subsequently, the virus enters in its target. Different roads of entry have been identified (Figure 1A). First, the most described, as aforementioned passes through ACE2 due to the RBD of the S protein [15,16,17]. This entry also implies the cleavage of the S protein, by the transmembrane protease serine 2 (TMPRSS2), which allows it to release the fusion peptide contained into the S2 domain and largely increases SARS-CoV-2 entry, similarly to what was shown for SARS-CoV [14,15,16]. Alternatively, a cleavage by the cathepsin L during endocytosis is also possible. Interestingly, alike the classical cleavage site known in the SARS-CoV S protein, SARS-CoV-2 S presents a furin-like cleavage site, often observed in highly virulent influenza viruses. This may permit cleavage in the Golgi apparatus during biosynthesis (the global structure still maintained by non-covalent bonds) and extend tropism and transmissibility, due to the near-ubiquitous expression of furin-like protease [14]. An abundance of literature demonstrated ACE2/RBD binding, but recently, a new route of entry has been evoked via CD147 (Basigin) and needs to be further explored [25], as these results are still questionable.

#### 2.2.2. Release and Replication

The process of entry and fusion lead to virion contents (i.e., nucleocapsid) release inside the targeted cell (Figure 1B). The next step in the viral cycle is the replicase–transcriptase complex translation directly from the genomic RNA (Figure 1C), a process requiring ribosomal frameshifting [10,26]. The encoded polyprotein, as said earlier, is processed to give 16 nsp, which assemble to form the replicase–transcriptase complex. In coronaviruses, this complex exerts the particularity to be associated with remodeled cellular membranes, only dedicated to welcome viral RNA synthesis [27,28], representing a mean of immune evasion. Afterwards, negative-sense RNA intermediates are generated to serve as templates for the synthesis of genomic RNA on the one hand, and subgenomic RNA on the other hand that will encode structural proteins and several accessory proteins (Figure 1E) [29].

#### 2.2.3. Assembly

After translation, M, S and E proteins are inserted in the endoplasmic reticulum (ER) membrane (Figure 1F). From this location, they are transported, through a secretion pathway, to the site of assembly: the endoplasmic reticulum–Golgi intermediate compartment (ERGIC) [10,30]. Assembly requires a complex cooperation between the M protein and other structural proteins, notably the E protein, which is essential for assembly [5,10,30,31]. N protein complexes with newly synthesized genomic RNA in cytoplasm to form NP, which then reaches the ERGIC to which achieves progeny viral formation by condensation of NP with the envelope components (Figure 1G). Conversely to what is known for entry and fusion of SARS-CoV-2, few data are available at this time about other steps of the viral life cycle. Hence experiments are highly awaited to address the direct cytopathic effects of the SARS-CoV-2 replicative cycle on cells that support infection [32,33]. Of note immune-induced tissue injuries need to be taken into consideration and these will be discussed largely below.

#### 2.2.4. Budding and Transmission

Finally, the last step of the virus life cycle is virion exocytosis (Figure 1I). Virus budding appears to be done near the Golgi apparatus, presumably into smooth membranes of ERGIC [28]. This leads to two phenomena: transmission and viral spreading in other organs. Transmission is mainly described by person-to-person airborne transmission, via droplets or contact [34,35,36]. However, other routes of transmission are supposed (such as orofecal or ocular), as the virus can be found in the digestive tract [37,38] and ocular surface express ACE2 [39].

Even if data might be extrapolated from what is known from other coronaviruses, going forward remains crucial to confirm them with a cellular model of infection.

### 2.3. Tropism and Associated Clinical Manifestations

**Figure 2:**
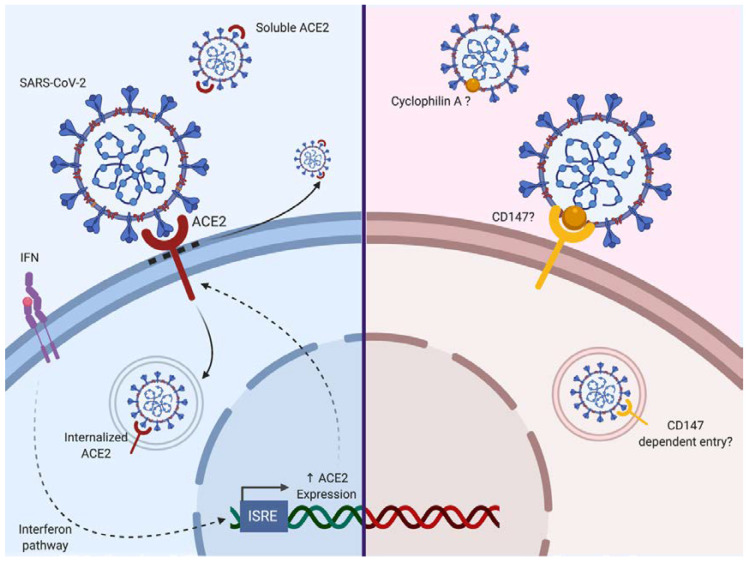
The two evoked routes of entry for SARS-CoV-2 to date. Angiotensin-converting-enzyme 2 (ACE2), which has been described as an interferon-stimulated gene (ISG), is a route of entry for SARS-CoV-2. Additionally, CD147 is evoked as a potential second route of entry. Based on a previous study with SARS-CoV, an interaction with Cyclophilin A is possible. The blue background corresponds to cells expressing ACE2, whereas the red background is representing cells expressing CD147. Solid arrows correspond to a direct activity involving ACE2, dotted arrows correspond to an indirect promoting activity.

As we have seen, SARS-CoV-2 is mainly supposed to enter via a principal surface protein ACE2 (and maybe other entry receptors/co-receptors such as CD147) and fuse via TMPRSS2 or furin (Figure 2). These proteins are expressed on several cell types and tissues, explaining the multiple organ injuries that can be observed in SARS-CoV-2 infection.

ACE2 is a carboxypeptidase responsible for the conversion of angiotensin II (a potent vasoconstrictor) to angiotensin (1–7; a vasodilator). ACE2 is expressed in the nose, lung, ileum, heart, eye, liver, bladder, kidney, pancreas, brain, prostate, testis and placenta [40,41,42]. Following binding of the SARS-CoV-2 S protein, ACE2 activity and its surface expression are decreased. Thus, the loss of ACE2 expression on the cell surface may create an unbalance in the renin–angiotensin system, resulting in reduced metabolization of angiotensin II and leaky pulmonary blood vessels associated with severe acute lung injury through Angiotensin II Type 1a receptor stimulation (AT1R) [43,44]. Additionally, it has been shown that the injection of recombinant SARS-CoV S protein itself in acid aspiration-induced acute lung injury in the mouse is sufficient to boost lung failure [43]. It may be that similar effects of SARS-CoV-2 S protein could be envisaged. It has been shown that SARS-CoV binding to ACE2 could lead to ACE2 shedding as a soluble form by ADAM17, also known as a tumor necrosis factor alpha (TNF-α) converting enzyme [45,46,47,48]. Inflammatory cytokines such as TNF-α could enhance ACE2 shedding [45,46]. Of further note, it is known that AT1R directly upregulates nuclear factor-κB (NF-κB) [49,50,51] and ADAM17 [52], which in turn can cleave membrane-bound TNF to produce soluble TNF. So, we may have here a vicious spiral where ACE2 loss leads to overstimulation of the AT1R axis and subsequent upregulation of NF-κB and ADAM17, triggering ACE2 shedding enhancement. TMPRSS2 can also participate in the processing of ACE2 [53]. Soluble ACE2 remains biologically active and competes for S protein binding. Furthermore, endocytosis of ACE2 after binding to SARS-CoV has been reported [54]. Interestingly, it has been reported that ACE2 is an interferon-stimulated gene in vitro, suggesting that the cell inflammatory response could indirectly enhance SARS-CoV-2 infection [55].

Other receptors have been reported to mediate SARS-CoV infection. DC-SIGN and L-SIGN serve as alternative receptors for SARS-CoV entry independently of ACE2 [56,57,58].

CD147, a surface transmembrane glycoprotein could represent a co-receptor for viral entry as it can enhance infection by SARS-CoV as well as HIV-1 or Measle Virus [59,60,61]. Chen et al. showed that N protein of SARS-CoV bound to Cyclophilin A could interact with CD147 [59]. This protein (also known as Basigin) is involved in the expression of matrix metallopeptidases and can interact with several proteins such as integrin, monocarboxylate transporter proteins, cyclophilins or caveolin-1 [62]. Cyclophilin-CD147 interaction has been involved in the regulation of inflammatory responses and the pathogenesis of various inflammatory-mediated diseases [63]. However to date, there is no evidence for CD147 as a direct SARS-Cov-2 binding receptor [64] although the Cyclophilin inhibitor Alispovir could inhibit SARS-CoV2 in vitro [65].

Vimentin a structural protein may also serve as a co-receptor as it can interact with SARS-CoV S protein during viral entry [66]. Additionally, the cellular receptor neuropilin-1 (NRP1) expressed on the olfactory epithelium has been shown to facilitate SARS-CoV-2 infection [67,68]. The interaction between the S protein and NRP1 may provide a possible pathway for virus entry into the nervous system through the olfactory system.

**Figure 3:**
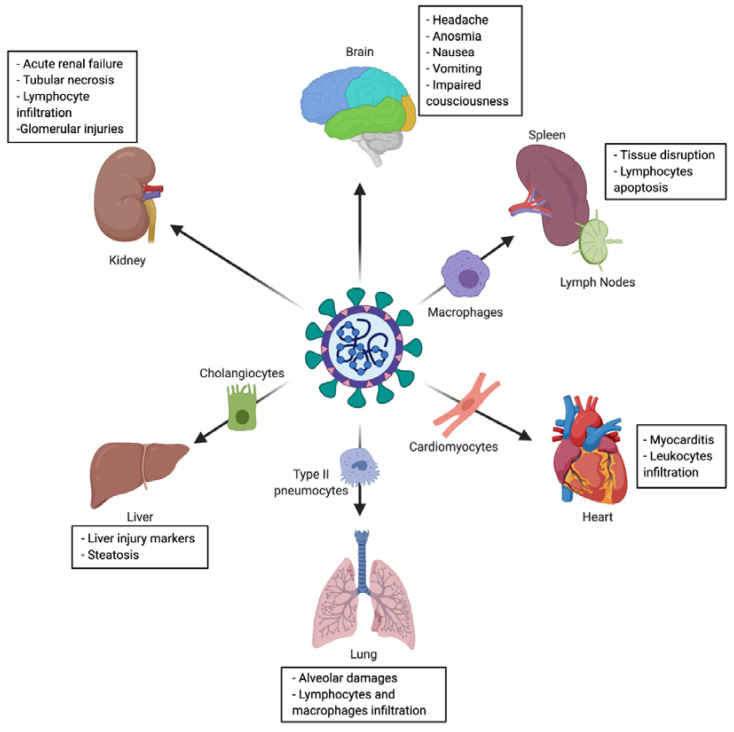
Tropism and multiple organ injuries in SARS-CoV-2 infection. SARS-CoV-2 infection has been associated with multiple organ injuries due to viral tropism. Among injured organs (and targeted cell) we can find: lung (type II pneumocyte), heart (cardiomyocyte), liver (cholangiocyte), spleen and lymph nodes (macrophage), kidney and brain.

The virus main infected cell-types and target-organs are described in Figure 3, and detailed hereafter.

#### 2.3.1. Nasal Cavity

Since SARS-CoV-2 is notably transmitted by droplets, the nasal cavity is its first route of entry. Among the cells present in the nasal cavity, respiratory epithelial cells (goblet cells and ciliated cells) were demonstrated to express ACE2 and TMPRSS2 by some [15,17,42] while only TMPRSS2 by others [69]. Thus, these cells represent an accessible source of viral material for detection of SARS-CoV-2 infection with higher viral load on the nasal swab than on the throat swab [70], meaning that the virus actively replicates in this place. Therefore, the nasopharyngeal swab is usually employed in routine for a diagnosis purpose. In addition, these data make these cells a possible reservoir for dissemination within and between individuals [42].

#### 2.3.2. Olfactory Epithelium

From a clinical point of view, nasal infection of SARS-CoV-2 is associated with olfactory and gustatory dysfunctions, which should be recognized as important symptoms of infection [71], observed in more than 50% of the patients [72,73]. Olfactory epithelium was shown to express ACE2 and TMPRSS2 [74], but also NRP-1 [67,68]. Even if pathophysiology underlying anosmia and gustatory dysfunction is not clearly elucidated, three mechanisms are evoked [75]. First, local inflammation due to infection of vascular and support cells of olfactory bulb, provoke downstream effect blocking odor conduction. Second, damage of support cells, responsible for water and ion balance, could influence signaling from olfactory sensory neurons. Third, damage to sustentacular and Bowman’s gland cells may lead to olfactory sensory neurons death and disruption of the entire olfactory epithelium [75].

#### 2.3.3. Lung

Lung is the organ the most concerned by SARS-CoV-2 outcomes. The most common symptomatology of COVID-19 is related to lung and respiratory tract involvement: dry cough, sputum production and shortness of breath). Indeed, infection leads to pneumonia (abnormalities on chest CT) and in severe cases to respiratory failure, which is generally the cause of death [2,9,76,77,78]. However, the COVID-19 pneumonia is an atypical viral pneumonia, with frequently observed dissociation between patients’ well-preserved lung mechanics and the severity of hypoxemia [79]. This discrepancy could be explained notably by lung vascular injury and thrombosis induction leading to lung hypoperfusion [80]. On a histopathological point of view, pulmonary changes are the most significant, although nonspecific. In the context of SARS-CoV-2 infection, different histopathological patterns of acute lung injury have been described so far: diffuse alveolar damage (DAD), which is closely associated with acute respiratory distress syndrome (ARDS), acute fibrinous and organizing pneumonia (AFOP) and lymphocytic pneumonia [81,82,83]. However, the frequency, the context and the clinical implication of various histopathological patterns is yet-to-be elucidated. DAD pattern, characterized by numerous hyaline membranes, is often reported, associated with lymphocytes and few macrophages infiltration, as congested alveolar capillaries [84,85]. Type II pneumocytes are the principal target of SARS-CoV-2 in the lung, leading to desquamative and dysmorphic pneumocytes (multinucleated syncytial cells, atypically enlarged, large nuclei, amphophilic granular cytoplasm and prominent nucleoli) [82]. The main pulmonary pathologic findings are summarized in Table 1.

Loss of ACE2 activity after infection has been suggested to be associated with acute lung injury as it induces an imbalance of the renin–angiotensin system as aforementioned [86].

#### 2.3.4. Gastrointestinal Tract

It is also possible to cite gastrointestinal outcomes including diarrhea, nausea, vomiting and rarely abdominal pain [87]. SARS-CoV-2 was reported to infect ACE2+ glandular cells in the gastric, duodenal and rectal epithelia [88]. Histopathology indicated infiltration of plasma cells and lymphocytes and interstitial edema in the lamina propria, in the context of SARS-CoV-2 infection [85,88]. Moreover, in some reports, infection in the gastrointestinal tract is associated with partial epithelial degeneration, necrosis, shedding of gastric and intestinal mucosa [85] and in other, with no damage of the mucous epithelium [88] (Table 1). Of note, if discharge criteria of patients are mainly based on the resolution of pulmonary symptoms (with resolved respiratory symptoms, improved acute exudative lesions on CT images and two consecutively negative RT-PCR from respiratory samples), gastrointestinal persistence of SARS-CoV-2 may be underrated. Indeed, viral RNA have been detected in stools of some patients even after resolution of respiratory symptoms and up to 35 days after onset of symptoms, leading to a protracted form of disease characterized by gastroenteritis symptoms requiring readmission to hospital [89].

#### 2.3.5. Liver

It is largely reported that aspartate transaminase (AST), alanine transaminase (ALT) and bilirubin levels are increased during COVID-19, these three markers testifying from liver injury. In addition, they are often accompanied by an increase of acute phase inflammation markers (procalcitonin and C-reactive protein) [2,76,78]. This liver injury is usually mild and transient. MERS-CoV was already associated with liver injury, confined in portal tract and hepatic lobule [90]. These findings were corroborated for SARS-CoV-2, with mild microvesicular steatosis, mild lobular and portal activity and centrilobular sinusoidal dilatation [81,82] (Table 1). It has been shown that SARS-CoV-2 is able to target cholangiocytes. Its cytopathogenic effects might in part explain the liver damages observed in COVID-19 patients, due to accumulation of bile acid [91]. Apart from viral direct effects, other mechanisms cannot be excluded, such as drug toxicity.

#### 2.3.6. Cardiovascular System

Patients with pre-existing cardiovascular disease are at higher risk for mortality from COVID-19 [92]. Moreover, cardiovascular complications are frequent among patients with COVID-19, regardless of their pre-existing conditions [93,94]. Myocardial inflammation and injury are a common manifestation of COVID-19 as it has been reported during the MERS-CoV or the SARS-CoV [95,96]. SARS-CoV-2 could infect and induce cytotoxicity in human cardiomyocytes in vitro and recent autopsy evidences confirmed that direct myocardial tissue infection might occur given that ACE2 is highly expressed in myocardium [97,98]. On a histopathological plan, some described cardiomyocyte hypertrophy, degeneration and necrosis of some cardiomyocytes, mild interstitial hyperemia, oedema, infiltration of a small number of lymphocytes, monocytes and neutrophil [85] while others reported a few interstitial mononuclear inflammatory infiltrates [82].

The endothelium is also an essential contributor to the pathogenesis of COVID-19 [99]. Histopathology indicated that SARS-CoV-2 might induce endotheliitis in several organs, including the lung, as a result of both direct endothelial cells infection and overreacting inflammatory response [100]. Moreover, endotheliopathy characterized by increased thrombotic and microvascular injuries are also an important feature of COVID-19 complications and are likely to be associated with critical illness and death [101].

#### 2.3.7. Brain

Regarding the proximity of nasal mucosa, the first entry-site of infection, with the brain (via the cribriform plate of ethmoid), it is possible to assume an infection of the central nervous system (CNS). Above all, CNS manifestations were reported during with previous epidemics of SARS-CoV and MERS-CoV, where CNS manifestations were reported [102,103]. Indeed, SARS-CoV had shown the ability to infect neurons [104,105]. Consistent with these data, CNS manifestations of COVID-19 are also depicted (headache, nausea and vomiting) suggesting that CNS is targeted by SARS-CoV-2. Among severe patients, even acute cerebrovascular diseases and impaired consciousness was observed [106,107,108]. However, these manifestations are clearly nonspecific, thus involvement of CNS remains to be ascertained, notably through cerebrospinal fluid explorations or autopsies [109]. Anyway, it remains that more specific neurological manifestations were observable in some patients [108]. Thus, Moriguchi et al. reported a first case of meningoencephalitis. This patient initially presented a generalized seizure and Glasgow score of 6 associated with SARS-CoV-2 RT-PCR positivity in cerebrospinal fluid but not the nasopharyngeal swab. Additionally, SARS-CoV-2 can lead to spinal cord injury and myelitis [107,108], which manifested as the Guillain-Barré syndrome notably in two case reports [110,111]. Finally, a possible involvement of SARS-CoV-2 infection in the development of long-term neuropathology, such as multiple sclerosis, is conceivable [108]. Indeed, SARS-CoV infection has already been linked with multiple sclerosis [108].

#### 2.3.8. Kidney

Acute kidney failure was reported during SARS-CoV-2 infection [112,113]. Among the clinical manifestations of kidney dysfunction, hematuria [114], elevation of serum creatinine [115], blood urea nitrogen [115] and/or proteinuria [114,115,116] were reported. SARS-CoV-2 seems to be able to target the kidney: virus-like particles were visible in the kidney by electron microscopy and immunohistochemistry showed accumulation of SARS-CoV-2 antigen in kidney tubules. Moreover, pathologic findings include diffuse proximal tubule injuries (loss of brush border, vacuolar degeneration and even acute tubular necrosis), lymphocyte infiltration [112] and glomerular injuries [85] (Table 1).

#### 2.3.9. Secondary Lymphoid Organs

On a whole new level, SARS-CoV-2 is targeting the immune system. Indeed, ACE2 was shown to be expressed by macrophages from lymph nodes and spleen. This has been related to histologic disruption of these secondary lymphoid organs [85,117]. Furthermore, even if T cells and B cells are not directly targeted by SARS-CoV-2, it has been shown that SARS-CoV-2 induces lymphocytes apoptosis via enhancement of Fas-signaling and IL-6 signaling, which may partially explain lymphopenia observed in infected patients [117], associated eventually to other cytokines-induced phenomena [118,119].

#### 2.3.10. Adipose Tissue

Recent data points to overweight and obesity as major risk factors for developing a severe form of COVID-19. In a recent French retrospective study nearly half of the patients admitted in an intensive care unit for COVID-19 had a body mass index—BMI over 30 kg/m^2^. In that study severe obesity was a risk factor to require invasive mechanical ventilation independently of diabetes, hypertension, sex and age [120]. These data are not surprising as obesity has been previously reported as an independent factor for severe respiratory form and morbidity in other viral infections such as H1N1 influenza [121,122].

A link between obesity and COVID-19 severity may be related to the adipose tissue. Adipose tissue can be a direct target of SARS-CoV-2 as the ACE2 receptor is found in adipocytes smooth muscle cells and endothelial cells within this tissue [123]. However, there is currently no evidence that the adipose tissue acts as a reservoir for the virus.

Adipose tissue is a highly vascularized and active secreting organ interfering with metabolism but also immunity. Obesity is considered as a low-grade inflammatory disease due to the altered profiles of cytokines secreted by the dysfunctional adipose tissue [124]. Obesity is associated with increased levels of proinflammatory cytokines secreted by adipose tissue as IL-6, IL-1 and TNF-⍺ and a decrease of anti-inflammatory cytokines such as IL-10. The “pre-activated” adipose tissue may participate in the explosive proinflammatory state in case of extensive infection. Severe forms of COVID-19 are associated with a “cytokine storm”, many of the reported cytokines are secreted in other inflammatory conditions by adipose tissue [125]. Lymphocytes and macrophages dysfunction are common in obesity with decreased Treg and lymphocytes T suppressor/helper. The same features have been found in severely ill patients with SARS-CoV-2 [125].

#### 2.3.11. Musculoskeletal Symptoms

Frequent myalgia and fatigue (44–62%) were reported in Chinese and European series [126,127]. The cell receptor gene ACE2 was also shown to be expressed at low levels in human muscular tissue [128]. Respiratory muscles might also be affected [129]. No joint or bone involvement was reported so far.

Even with all these data, fundamental research is urgently needed to assess outcomes of the infection in the different organs, as no consensus exist for organ injury, and knowing that some histopathological findings cited may be linked either to a direct effect of virus, to an exacerbated immune response, to pre-existing injuries or even to pharmacological care.

## 3. Immunopathology of SARS-CoV-2

### 3.1. Cellular Response

#### 3.1.1. Innate Immune Response

##### Pattern Recognition Receptor

**Figure 4:**
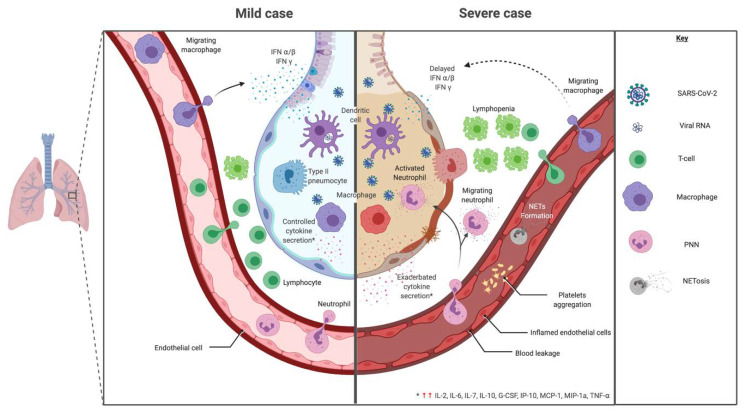
Mild versus severe immune response during SARS-CoV-2 infection. In regards to cytokine signature during SARS-CoV-2, mild and moderate cases showed a controlled response with higher expression of IL-1β, IL-1RA, IL-2RA, IL-6, IL-7, IL-8, IL-9, IL-10, basic FGF, G-CSF, GM-CSF, HGF, IFNγ, IP-10, MCP-1, MIP-1a, MIP-1b, PDGF, TNF-α and VEGF. While, a cytokine-induced immunopathological mechanism has been observed with an increase of IL-2, IL-7, IL-17, IL-10, MCP-1, MIP-1a and TNF-α in severe cases, leading to a bystander effect.

Innate immune cells need to recognize pathogens (via pathogen associated molecular pattern—PAMP) by receptors like pattern recognition receptor—PRR. In coronaviruses, as RNA viruses, endosomal receptor Toll Like Receptor (TLR) 3 and TLR7 and cytosolic retinoic acid-inducible gene I (RIG-I) and melanoma differentiation-associated protein 5 (MDA-5) have been shown to recognize SARS-CoV and MERS-CoV. This leads to activation of a signaling cascade through NF-κB and IRF3 pathways, resulting in a classical immune antiviral response with induction of type I interferon (IFN) and other proinflammatory cytokines as shown in Figure 4 [130]. During SARS-CoV and MERS infection, diverse dampening strategies of this cytokine response have been shown and are related to disease severity [131].

This immune evasion leads to a greater viral load by delaying the type I IFN response thus following in an exacerbated bystander immunopathogenicity [132,133]. One key component of the immunopathogenesis is the accumulation of macrophages and neutrophils in tissues.

SARS-CoV-2 seems to follow the same pathways for recognition and dampening strategies, but other mechanisms may be discovered [130,134]. The major point is the suppression of the type I IFN response [130,135,136]. Although monocytes/macrophages and dendritic cells should theoretically be key producers of type I IFN, as primary responding tissue innate immune cells, studies to date showed minimal cytokine expression in the first stages of the disease.

##### Dendritic Cells (DCs)

As the strongest antigen-presenting cells (APC), dendritic cells (DCs) play a leading role in stimulating and linking innate and adaptive immunity. They represent one of the first-line cells during the onset of infectious disease. Thus, they are a direct target of viral evading strategies to reduce the immune response. Studies on DCs susceptibility for other coronaviruses showed susceptibility to SARS-CoV but at a low level and without a strong triggering of type I IFN secretion. On the other hand, MERS-CoV was able to activate DCs and to induce a greater cytokine reaction than SARS-CoV [137]. With the presence of ACE2 on the surface membrane of DCs, a question remains on their infectability by SARS-CoV-2 [138]. As major producers of type I IFN, they also play a role in cytokine release and attraction of immune cells. Comparably to SARS-CoV, the induction of such cytokine during SARS-CoV-2 infection is to be further explored [134]. In COVID-19 patients, DCs percentage in blood did not change with disease severity but they showed a reduction of CD86 expression in severe compared to mild disease [139]. Variation of DC subsets has been observed. CD1c+ DCs were decreased with disease severity and recruited in the lung during ARDS [140].

##### Macrophages

Monocytes and macrophages are important innate immunity actors of airway interface. In pulmonary infection, alveolar macrophages initiate the production of type I IFN [141].

Alveolar macrophages express ACE2 but at a low level [138]. They are present since the initiation of the disease and represent one of the initial cells involved in the innate immune response. The mechanism of entry of the virus in macrophages is still under study: through phagocytosis, ACE2-dependent [117] or -independent pathways. Studies of the SARS-CoV and MERS-CoV cellular response showed a delayed cytokine response in the initial stages of the disease. Later, they are responsible for releasing high levels of proinflammatory cytokines but low amounts of IFNs. However, the IFN response in the initial stages of viral infection is key to mounting antiviral immunity. The secondary rapid cytokine release by recruiting proinflammatory cells is hypothesized to be responsible for host tissue injury [142]. During COVID-19, a decrease of classical monocytes (CD14+ and CD16−) was observed with an increase in intermediate (CD14+ and CD16−) and non-classical (CD14− and CD16+) monocytes [141]. A mouse model of SARS-CoV infection showed that a delayed type I IFN release increased disease mortality through accumulation of inflammatory macrophages [143]. Depletion of those macrophages, but not of neutrophils, led to a protective effect on disease mortality without an increase in viral load [143]. In bronchoalveolar lavage of COVID-19 infected patients, monocytes-derived macrophages (overwhelming tissue resident macrophages) and neutrophils were increased in severe patients. Additionally, macrophages had an M1-like gene expression pattern in severe patients whereas moderate patients had a more M2-like macrophage profile [144]. Type I IFN can induce macrophages polarization depending on their microenvironment [141]. During severe COVID-19, macrophages have a M1 proinflammatory cytokine profile and are likely to be a key factor of cytokine related severity clinical features [145,146].

##### Polymorphonuclear Neutrophils (PMN)

Neutrophils are predominant actors for disease control in innate immunity specifically during respiratory diseases and ARDS. Neutrophils have a central role in driving an inflammatory pathogenic state, with the production of reactive oxygen species (ROS), cytokines, proteases and neutrophil extracellular traps (NETs), which exude histones and DNA [147,148,149]. NETs are key features to contain infection but some pathogenic effects have also been described [150,151]. As ARDS and immunothrombosis are predominant features in critically ill patients in COVID-19, the implication of neutrophil in this setting has to be explored. In previous coronaviruses infections, neutrophil appears as a major actor of aggravation rather than disease control. An animal model of depleting neutrophil during coronavirus infection shows an essential role of PMN for mounting an effective viral response but induced lung injury [152]. An increase in neutrophil count has been associated with disease severity. Single-cell RNA sequencing based peripheral blood mononuclear cells profiling showed an increased neutrophil subpopulation expressing a progenitor profile in severe cases consistent with the reported neutrophil expansion [153]. Furthermore, the reduction of the ratio of neutrophil to lymphocyte is associated with a worse outcome [154]. A recent study of NETs during COVID-19 showed an increased sera level of cell-free DNA, myeloperoxydase-DNA and citrullinated histone H3. These were increased in critical patients needing mechanical ventilation compared to those not mechanically ventilated [155,156]. This role of NETs in ARDS may represent a new therapeutic target in those settings [148,155]. At a histological level, neutrophil infiltration has been confirmed in autopsy samples [155].

##### Complement

The complement system plays an essential role during response to bacterial, viral and fungal infection. It acts rapidly to activate the host immune system and attract cells towards the infection site. Its activation is tightly controlled to avoid detrimental injury. C3a and C5a have proinflammatory properties by recruiting inflammatory cells and activating PMN. They participate in acute pulmonary injury during coronavirus infection [157]. The use of an anti-C5a antibody in a mouse model of MERS-CoV infection has shown an improvement of respiratory symptoms without an increase in viral load [157].

A recent histological study of pulmonary and cutaneous biopsies or autopsy samples from five critically ill patients infected by SARS-CoV-2 showed complement mediated microthrombotic disease with deposits of C5b-9, C4d and Mannan-binding lectin serine protease 2 (MASP2). Thus, some atypical ARDS features of COVID-19-related severe respiratory distress might be caused by microvascular injury after activation of complement pathways [158]. The complement system seems a good candidate for a therapeutic target to limit disease severity by limiting inflammatory cell recruitment or direct complement activation related microthrombopathy [134,158].

##### Natural Killer (NK) Cells

With CD8+ T cells, NK cells are predominant actors to impeding viral replication by killing infected host cells. In previous studies on SARS-CoV, a dampening of cell-mediated cytotoxicity was observed with a reduction of NK and CD158+NK cells with disease severity [159]. Animal models of SARS-CoV infection with specific NK cell depletion found no aggravation of the disease suggesting that NK cells are not essential for the SARS-CoV immune response [102,159]. Clinical studies during COVID-19 showed a decrease of NK cell count in severe but not mild disease, probably linked to viral propagation [146,153,154]. The dynamic profile of the NK cell count did not show differences between dead or survivor groups of the most severe patients [160]. The time to NK cell normal cell count recovery paralleled clinical amelioration. However, SARS-CoV severe disease was marked by a functionally exhausted profile with increased membrane expression of NKG2a and a reduction of CD107a+, IFNγ+, IL-2+ and TNF-⍺+ NK cells [161]. Wilk et al. demonstrated that NK CD56+ cells were reduced in all COVID-19 patients and NK CD56- cells were reduced in ARDS patients [153].

#### 3.1.2. Adaptive Immune Response

##### T Cells

Secondarily to the innate immune cells initial reaction, the adaptive immunity plays an important role in recognition and killing of infected cells with cytotoxic CD8+ lymphocyte and in mounting a humoral antigen specific response with CD4+ lymphocyte. Dysregulation of lymphocyte subsets can occur during viral infection. During COVID-19, lymphopenia is a common feature [162]. A decrease of total lymphocytes but also of CD4+ T cells, CD8+ T cells and B cells decrease was reported. The lymphocyte levels were lower in severe compared to mild cases. This is comparable to data during SARS or MERS infection. In addition, CD8+ T-cell decrease and CD4/CD8 ratio increase were correlated with disease severity [162]. The normalization of the different subset cell counts appeared during the clinical convalescence.

In the context of previous coronaviruses epidemics, a strong T-cell response was associated with higher titers of neutralizing antibodies. Specific T-cytotoxic cells were still found 10 years after infection with SARS or MERS infections [163,164,165,166].

During SARS infection, the viral structural proteins E [167], S [168], M [169] and N [167,170] were shown to induce T cell antigen-specific responses [165,166]. The acute phase is followed by an important contraction of the antigen-specific T cell pool. T CD4 antigen specific cells presented a central memory phenotype (CD45RA− CCR7+ CD62L−) with production of IFNγ, TNF-⍺ or IL-2 whereas CD8+ T cells presented an effector memory one (CD45RA+ CCR7− CD62L−) with production of IFNγ, TNF-⍺, perforine and granzyme [59,165,166]. Emerging data point that memory CD4+ T cell specific for commonly circulating human coronaviruses (HCoV-OC43, HCoV-229E, HCoV-NL63 and HCoV-HKU1) can cross-react with SARS-CoV-2 epitopes [171]. Thus, Mateus et al. mapped 142 T cell epitopes across the SARS-CoV-2 genome and showed a population of responsive memory CD4+ T cells, even in unexposed individuals [171]. Even if it is still very speculative, this pre-existing repertoire of memory CD4+ T cell against other coronaviruses may cross-react with SARS-CoV-2 and contribute to variations observed in COVID-19 patient disease outcomes [171,172,173]. This hypothesis is supported by similar data, indeed, reactivity in blood samples of unexposed individuals has been reported by several teams [174,175,176,177,178].

##### B Cells

The importance of mounting the humoral response was shown by the presence of a non-switched B lymphocyte profile in deceased patients from SARS or MERS viruses. The role of neutralizing antibodies is essential in a later stage of the disease and to prevent reinfection.

Evaluation of the antibodies response after SARS-CoV infection showed apparition of IgG as soon as 4 days after infection. Most patients had a seroconversion at 15 days [163,179]. Antibodies directed against the coronavirus S protein are protective neutralizing antibodies but binding non-neutralizing antibodies against other viral proteins are also described [180]. During the follow-up period, IgM peaked at 1 month and IgG at 2–4 months [181]. A decrease of antibody titer was observed at 3 years [181] and undetectable in 21 of 23 patients at 6 years [182]. There was no association between the drop of neutralizing antibody titer and disease severity, comorbidity or treatment use [183].

In SARS-CoV-2, the same chronological appearance of IgM and IgG has been observed [184]. In severe cases, a strong acute humoral response level has been reported [185] with increased proportion of plasmablasts in COVID-19 patients [153]. Nevertheless, a rapid decrease of the SARS-CoV-2 specific antibodies titers has been demonstrated in severe cases [186,187,188]. Variation of antibody response kinetic with disease severity [188,189], anti-N and anti-S antibodies antigen specificity [187,189], cytokine environment variation or major lymphopenia with disease severity [190] has been described. Their impacts on humoral protection maintenance have to be further studied. Long-lived plasma cells and memory-B cells responses and maintenance are important actors of long-term humoral protection [191,192]. The mechanisms underlaying the lack of long-term humoral immunity in some infectious disease are poorly understood [193]. Pathogen’s antigen evasion mechanism [194], lower B-memory cell response [195,196] or long-lived cell homeostasis anomaly [197,198] may be leads to explore.

In some animal and human coronaviruses, the mechanism of antibody-dependent enhancement (ADE) has been suspected comparably to the dengue virus. Notably for SARS-CoV, the in vitro experiment showed enhanced infection with specific anti-spike antibodies highly diluted [199,200]. Based on the similarity between SARS-CoV and SARS-CoV-2, some question the possibility of occurrence of ADE during COVID-19 [201] but to date no strong evidence has been found [202].

Post-infectious autoimmune disorders have been reported [203,204] such as antiphospholipid syndrome [205], immune thrombocytopenic purpura [206,207], autoimmune hemolytic anemia [208,209], Guillain-Barré syndrome [110,210,211] or Kawasaki-like syndrome in children [212].

### 3.2. Molecular Response

To mount an effective antiviral response, innate and adaptive immunity are essential to control viral infection and clean infected cells with limited inflammation. For their actions immune cells release variable panels and levels of cytokines. This release if uncontrolled can have detrimental consequences for the patient. A delayed response may induce viral tissue injury, which can in turn lead to an exaggerated proinflammatory cytokine response. In the setting of sepsis and multi-organ dysfunction syndrome (MODS), two phases are described. An early phase with systemic cytokine release, neutrophil-generated ROS and cytotoxic lymphocyte inducing inflammatory systemic response with endothelial damage. A later phase consists of an amplification of inflammation by interstitial compartment cells in organs further exacerbating parenchymal injury [213].

The pulmonary failure during MODS appears as acute lung injury or ARDS (Figure 4). In pathogenesis, cytokines act to initiate and amplify the inflammatory response. Some major cytokines found are IL-1, IL-6, IL-8, IL-10 and TNF-⍺. The cytokine attraction and activation of neutrophils acts as the main pathogenic system inducing epithelial lung injury. This was confirmed with histological studies [213].

ARDS and immunothrombosis are predominant features in severe COVID-19 [142,214]. It has been long described the importance of neutrophil activation with NETosis, cytokine, ROS and protease release in pathophysiology of viral ARDS and immunothrombosis in sepsis [147,148,149,215,216].

In SARS-CoV infection, the viral 3a protein has been shown to induce NLRP3 mediated inflammasome activation [217]. A delayed innate immune response with low type I IFN release was associated with a later exacerbated proinflammatory (such as IL-1 and IL-6) cytokine response in severe cases. The timely cytokine response is necessary for a rapid recruitment and clearance of neutrophils and transition to adaptive immunity with minimal immune tissue damage [218].

In regards to cytokine signature during SARS-CoV-2, studies showed an adapted, controlled response in mild and moderate cases with higher expression of IL-1β, IL-1RA, IL-2RA, IL-6, IL-7, IL-8, IL-9, IL-10, basic FGF, G-CSF, GM-CSF, HGF (hepatocyte growth factor), IFNγ, IP-10, MCP-1, MIP-1a, MIP-1b, PDGF, TNF-α and VEGF compared to a healthy control as shown in Figure 4 [2,126,219]. These results are summarized and compared to other infectious settings in Table 2 [126,220,221,222,223,224].

In severe cases, clinical features show severe pneumonia, ARDS or MODS [2]. A cytokine-induced immunopathological mechanism has been observed with an increase of IL-2, IL-7, IL-17, IL-10, MCP-1, MIP-1a and TNF-α compared to moderate cases [126] (Figure 4). Other studies looking for cytokine biomarkers of worse prognosis between severe and moderate cases show that most cytokines were comparable between groups [2,219]. However, IP-10, MCP-3 and IL-1RA were significantly higher with a correlation to gravity evaluated by PaO2/FiO2 and Murray scores [219]. TNF-α, IL-1, IL-8 and IL-6 also increased in severe vs. non severe cases [220]. TNF-α has been shown to enhance ACE2 shedding, which participate to lung injury during SARS-CoV [145]. There was no increased risk of severe COVID-19 for patients treated by TNF-α blockers [225]. A case of severe COVID-19 disease in a recently diagnosed Crohn’s disease was ameliorated under TNF-α blockade [226]. Similarly to SARS-CoV, pyroptosis may be triggered during COVID-19 leading to major IL-1β production and uncontrolled cytokine release [145,217]. An increased IL-6/IFNγ ratio, found in severe cases, could be a sign of inappropriate immunopathologic cytokine response [218].

Those deregulatory cytokine features may represent potential therapeutic targets for severe COVID-19. The family of dysregulated cytokine syndromes is vast and often associated with critical life-threatening features. It is generally simplified as “cytokine storm” but comprise of a wide range of primary or secondary etiologies [227]. Familial hemophagocytic lymphohistiocytosis (HLH), macrophage activation syndrome (MAS) a secondary HLH complicating an underlying autoimmune disease, MAS-like sepsis, which appears in severe sepsis with MODS, are classical representatives of the “cytokine storm syndrome” [228].

In comparison to primary and secondary HLH, the viral immunosuppression during severe COVID-19 is supposed to arise from blood lymphopenia [126], NK and CD8 exhausted profiles [161] and interferon suppression [214,229]. Following the primary delayed response, a secondary excessive cytokine non-type I IFN but proinflammatory profile (IL-6, IL-1, IP-10, IL-18, GM-CSF and IFNγ) appears [146,214,219]. Furthermore, an increased and persistent viral release draws a cytokine and T-cell response to clear infection but induces, at the same time, pulmonary damage and ARDS. This offers explanation to clinical features typically found in severe COVID-19 pneumonia [214].

Some particularities of severe COVID-19 differ from HLH or MAS. The immune deregulation seems more confined to the lung with features of ARDS, which is linked to hypercytokine release by macrophages, attraction and activation of neutrophils [214,215,229].

The typical ARDS features found in severe COVID-19 has a cytokine profile comparable to H1N1(2009) infection where elevation of IL-6, IP-10 was observed in severe but not mild disease [230]. Compared to other pathological conditions (H1N1 influenzae and bacterial infection), patients with COVID-19 experiencing ARDS showed lower global severity, as assessed with lower SOFA score. This suggests an earlier respiratory failure in COVID-19 [146,231].

In many studies, sepsis is used to understand severe COVID-19 physiopathology. A study trying to classify COVID-19 patients into three immune states found during sepsis (MAS-like, dysregulation with immunoparalysis or functional) showed that all severe cases had an immune dysfunction with the majority of patients harboring dysregulated immunity while the rest presented MAS-like profile [146]. To confirm this classification, a specific severity score (H-score) was increased in MAS-like severe COVID-19 patients but not in the dysregulated group with immunoparalysis. In the latter group, serologic measures showed no measurable levels of IFNγ and an increase in IL-6 and CRP levels. IL-6 being one of the drivers of immunopathogenesis, a specific therapy was tested but showed only partial efficacy. Further studies need to be produced.

Another predominant cytokine driver of immunopathogenesis described during viral ARDS is IP-10 [229]. Chemoattractant cytokines for neutrophils overproduced by macrophages in lung injury are also found during severe COVID-19. They represent potential therapeutic targets for severe cases [219].

### 3.3. Immune Evasion

Coronaviruses are well armed to evade the immune system and particularly the initial innate immune response. The mechanism of molecular immune evasion is still to be confirmed for SARS-CoV-2 but the experience of previous SARS and MERS coronaviruses points some potential ways of immune evasion [130,232]. A common mechanism to avoid recognition of the virus is the formation of a double vesicle shielding from PRR sensing [232,233]. Among the structural proteins that serve in the architecture of the virion, SARS N protein is suspected to interfere with RNA recognition by the host immune sensors [232,234,235,236]. SARS and MERS protein M interact with TRAF3 to disrupt the TRAF3-TBK1 complex [232,237].

MERS non-structural protein 4a (NSP4a), 4b (NSP4b) and papain-like protease (PLpro) are described to have an immunity-evading function [232,234,237]. NSP4a interacts with PACT to inhibit dsRNA recognition and inhibit protein kinase R (PKR) [232,237,238,239]. NSP4b antagonizes OAS-RNAse L pathway thus limiting RNAse L activation and binds to TBK1 [232,237,240]. NSP15 also limits RNA sensor activation but through unknown pathway. PLpro protease uses deubiquitination to inhibit ISG15 action [237]. Additionally, NSP 1 in SARS and MERS degrade host mRNA [237,241,242].

**Figure 5:**
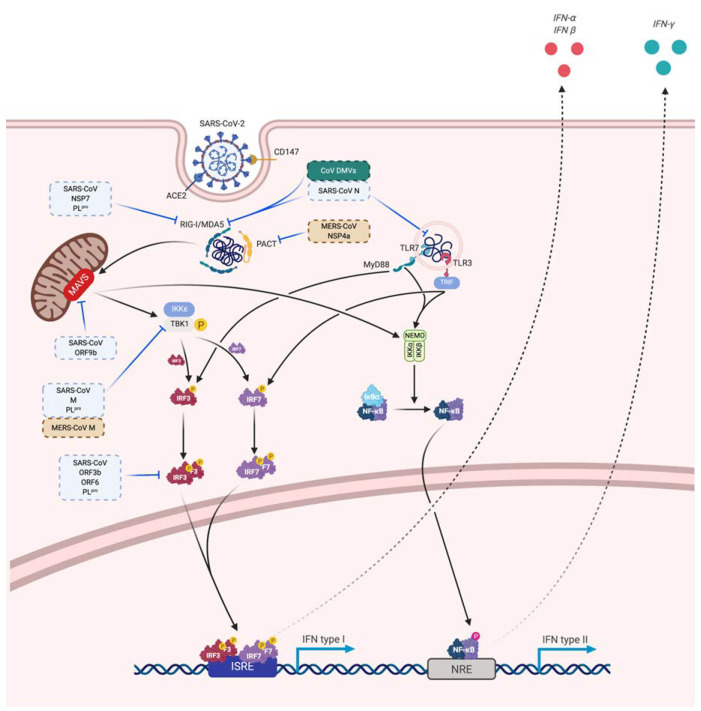
Viral sensing, innate antiviral response and immune evasion. Potential mechanisms of SARS-CoV-2 immune evasion based on previous studies on MERS-CoV (brown) and SARS-CoV (blue). Some mechanisms are inhibiting viral sensing, whereas others are directed against the innate antiviral response. Solid arrows correspond to a direct promoting activity, dotted arrows correspond to an indirect promoting activity and T-bars correspond to a direct inhibitory activity.

For SARS, NSP 7, 16 and 15 and accessory proteins ORF3b, ORF6 and ORF9b have also inhibitory activities. NSP16 prevents RNA sensing by MDA5 by formation of cap structures [243]. ORF9b acts by inducing a proteosomal degradation of MAVS/TRAF3/TRAF6 [244]. The others dampen IFN production through unclear mechanisms but ORF3b and ORF6 alter IRF3 activation and thus antagonize IFN production and signaling through IFNβ notably [232,234]. Furthermore, SARS-CoV PLpro inhibits IRF3 activation and deubiquitininate RIG-I, TBK1 and IRF3 [232,245]. Those potential mechanisms for SARS-CoV 2 immune evasion are summarized in Figure 5.

Other potential mechanisms are a reduction of presentation to an adaptive immune cell by downregulation of major histocompatibility complex (MHC) expression on antigen-presenting cells, viral mutation, immune cell exhaustion and immune deviation to the Th2 profile or localization in the immune privilege site [130,246].

## 4. Regards to Discrepancies in Diverse Immune Conditions

### 4.1. Elderly

During COVID-19 epidemics, studies identified age as a major risk factor of disease severity and mortality. A higher proportion of severe, critically ill patients or deaths appeared within this population [247]. The progression to severity was also shorter. Some symptoms (shortness of breath and lymphopenia) and comorbidities (cardiovascular disease, chronic obstructive pulmonary disease and acute respiratory distress syndrome) were predictive of worst outcomes [247].

An explanation to this excess mortality is the mounting number of comorbidities with age. A reduction of T-cell diversity appears with aging. Interestingly, naive T-cell diversity is strongly reduced after 65 years old [248]. However, modification of senescent T-cell profile appears to compensate this lack of diversity and replication with aging [248]. Lega S. et al. hypothesized that disease severity with aging may be due to immune senescence with an insufficient pool of specific T-cell to overcome the viral replication [249]. In regard to host immunity during COVID-19, the part of the immunosenescence lacking an antiviral response is to be further explored.

### 4.2. Children

Since the beginning of the epidemic, children seem to be spared from infection by SARS-CoV-2 [250]. In February, a Chinese cohort found only 2.4% of patients under 19 years old. Among these, they had 2.5% of the severe forms and 0.2% of critically ill patients, compared to 13.8% and 6.1% respectively in the whole cohort [251]. They appear to more often suffer from gastrointestinal symptoms [250]. The lesser potency in children was also observed during SARS-Cov and MERS disease [250]. The infection mostly occurs through household contact. The immune system shows some discrepancies between early and adult life in innate and adaptive immune cells. This results in a difference in response to the pathogen including viruses with some being more potent during infancy [252]. For SARS-CoV-2, the immune response seems favorable in childhood compared to adulthood.

Nevertheless, the appearance of a pediatric multisystem inflammatory syndrome has been reported with clinical features of myocarditis and Kawasaki-like disease after severe SARS-CoV-2 infection [212,253,254].

### 4.3. Pregnancy

In regard to infectious disease and especially respiratory illnesses, pregnant women represent a high-risk population. The same has been reported with SARS-CoV and MERS-CoV [255]. For SARS-CoV-2 infection, an increased risk for preterm delivery, preeclampsia, premature rupture membrane or stillbirth seem to appear [256] but the mother prognosis seems better than during SARS-CoV or MERS-CoV diseases [255]. Mortality and morbidity during pregnancy are still unclear.

Some specific elements may explain such over-risk. Firstly, some common gestational symptoms may mask initial, non-severe COVID-19 symptoms like rhinitis or dyspnea. Thus, leading to community transmission and late diagnosis. Furthermore, reduction of total lung capacity with advancement of pregnancy can predispose for respiratory failure [255].

Secondly, focusing on the immune system, there are changes in innate and adaptive immune responses in the face of an infectious challenge like strong NK cells or monocytes responsiveness [256] or a shift of the Th1 cytokine profile to a Th2 profile [255]. The pregnancy is associated with global viral susceptibility. It is to note that, differently to SARS-CoV, the cytokine profile during SARS-CoV-2 infection shows Th1 but also Th2 (IL-4 and IL-10) cytokine increase. The Th2 shift profile in pregnant women may thus serve to reduce severity [255]. Additionally, the cytokine release occurring during severe COVID-19, in addition to the gestation-linked proinflammatory state during the first and third semester, can alter fetal development and explain poor pregnancy outcomes [256].

In regard to their fetuses, no warning signal of vertical transmission has been observed [255].

### 4.4. Men/Women Susceptibility

A recent review of clinical and epidemiological findings during COVID-19 shows a 3-fold mortality increase risk in males compared to females [257]. This observation was also reported during SARS-CoV and MERS-CoV epidemics [258]. An animal model, with mice infected by SARS-CoV, reproduced this sex bias, which increased with age [258]. Sex hormones were associated to female protection.

In living organism, gender is described to influence an immunological response and affect innate and adaptive immunity alike [259]. It impacts apparition of autoimmune disease but also responses to infectious diseases. Furthermore, this influence changes over the course of life [259].

A recent review of current knowledge on sex difference of innate immune cells during respiratory virus infection showed a variety of receptors levels depending on cell subset and tissue localization [260]. Furthermore, they may act directly to promote or reduce cell function but also can act indirectly by modulating non-immune cell responses [260].

### 4.5. Genetic Susceptibility

Clinical aspects of COVID-19 disease are highly variable ranging from asymptomatic to life-threatening multi-organ failure disease. Differences in host genetic susceptibility may explain part of those differences in response to viral infection [261]. Host genetic polymorphisms have been associated with clinical severity in viruses [262,263]. During the SARS-CoV outbreak, HLA [264] and ABO [265] groups have been associated with disease severity. Using a genome-wide association study during the SARS-CoV-2 pandemic, HLA risk polymorphisms were described [261,266]. A susceptibility locus at chromosome 3p21.31 (spanning SLC6A20, LZTFL1, CCR9, FYCO1, CXCR6 and XCR1 genes) has notably been observed [266]. The ABO group seems to be implicated with a lesser risk of infection in the O group and higher risk in the A group [266,267,268]. Further studies may bring new genetic susceptibility association.

## 5. Immunotherapy

No antiviral therapy is available for treatment of COVID-19. Vaccines are under intensive development, and numerous clinical trials have been initiated, this could be the subject of a full-fledged review. For this reason, we will focus on SARS-CoV-2 management due to immunotherapy.

### 5.1. Antibody-Based Therapy

The first approach would be to use an old trick (i.e., serum therapy or serotherapy) invented by Emil Behring, more than 100 years ago, to prevent diphtheria. The purpose is to use serum from individuals who recovered from COVID-19 to treat suffering patients, the recovered serum containing neutralizing antibodies directed against SARS-CoV-2 creating passive immunity against the virus. Serotherapy, intravenous Ig (IVIg) or therapeutic plasma exchange (TPE) has been mentioned by several teams [269,270,271] and could be envisaged in prophylaxis or treatment, even if prophylactic injection or injection early after symptoms onset give better results than later injection [272,273]. Unlike vaccine therapy, serotherapy gives an immediate but short-lasting immunity (from weeks to months according to amount and composition) to susceptible persons. Specific antibodies are essential in viral clearance. Thus, convalescent plasma use may bring direct antiviral effect. Additionally, serotherapy has immunomodulatory effects that can be helpful in severe COVID-19 [274,275]. This solution has been used during the SARS-CoV outbreak and showed good results [276,277]. This therapy needs to be evaluated by clinical trials, taking in account all the challenges this practice could be facing [278]. Notably the potential risks could be: infection with another pathogen due to injection, reaction with serum components, ADE as a consequence of subneutralizing antibodies could thus facilitating entry of SARS-CoV-2 in cells [269]. Preliminary studies in severe cases show a reduction of mortality after convalescent plasma use [279] and need to be further explored.

Polyvalent IVIg, another blood derived product, does not contain neutralizing antibodies against SARS-CoV-2 and preparation/availability of specific IVIg from convalescent donor may take time [274,280]. Efficacy of IVIg during SARS-CoV has been variable [280]. Recent clinical trials seem to show an efficacy of high-dose IVIg in severe patients during COVID-19 [281,282]. Additionally, IVIg has been useful to treat autoimmune complication due to immunomodulatory effects [283,284].

As shown in sepsis, plasmapheresis was evoked as adjunctive treatment in severe COVID-19 [271,285] to control the dysregulated immune response but its efficacy is still under question [286,287]. A recent case series showed improved outcomes after plasmapheresis [288]. Additionally, it has been proposed to use convalescent plasma for a better efficacy of plasmapheresis [289]. Therapeutic evaluation through randomized trials is still needed.

Another aspect of antibody-based therapy could be the use of recombinant antibody targeting spike protein of SARS-CoV-2. Several antibodies have been described to target SARS-CoV-2 S. CR3022, which was initially isolated from a SARS-CoV convalescent patient showed ability to cross-react with a highly conserved cryptic epitope of S [20]. 47D11 showed the same ability [290]. Additionally, immunoglobulin fragment F(ab’)2 against RBD produced in horses exhibited a potent capacity to neutralize SARS-CoV-2 in mice [291]. Similarly, 2B04 a murine monoclonal antibody that binds RBD showed a potent neutralization of SARS-CoV-2 on the mouse model of infection, reducing weight loss and viral burden [292]. Of further note, a recombinant extracellular domain of ACE2 fused to the Fc region of human IgG1 (called ACE2-Ig) showed high affinity for SARS-CoV-2 RBD, and could be considered for treatment [293]. Interestingly, Gammunex^®^ and Flebogamma^®^, which are currently available polyvalent IVIg, showed cross-reactivity with different coronaviruses, including SARS-CoV-2 [294]. Recently, the risk of escape mutant generation was raised under individual Spike-specific antibody selective pressure in vitro. This was prevented due to an antibody cocktail, against two non-overlapping regions of SARS-CoV-2 spike protein, which represents a therapeutic avenue limiting emergence of escape mutants and subsequent loss of drug efficacy [295].

### 5.2. Immunomodulators

A viable therapeutic treatment against COVID-19 is urgently needed. Drug repositioning is a fast way to produce therapeutic treatment. Numerous trials assessing antiviral or immunomodulatory efficacy of different treatments are ongoing [296].

An initial focus has been made on hydroxychloroquine, a derivative of chloroquine. Hydroxychloroquine is a known antimalarial and immunomodulatory molecule actually used for treatment during autoimmune diseases such as systemic lupus erythematosus (SLE). It acts by increasing pH in endosomes thus interfering with the antigen processing of antigen presenting cells (APC) [297]. An antiviral effect of chloroquine on both entry and post-entry stages of SARS-CoV-2 infection has been described in vitro, which could add to its immunomodulatory effect in vivo [298]. Potential mechanisms are not fully understood. Presumably, the increase of pH in endosomes and drug accumulation in lysosomes prevent viral entry and transport [299]. Furthermore, an antiviral action during SARS-CoV infection was shown by interfering with the terminal glycosylation of ACE-2 [300] and glycosylation of viral particles [301]. A review of clinical trials and cohort study of hydroxychloroquine showed conflicting results on efficacy and viral clearance [302,303,304]. Adverse events of hydroxychloroquine are long known. The most serious adverse events are ocular (retinopathy) following chronic exposition, cardiovascular (restrictive or dilated cardiomyopathy and conduction abnormalities), cutaneous (alopecia, pruritus, photosensitivity and skin eruption), neurologic (headache, dizziness, vertigo, convulsion and polyneuropathy), hepatic (abnormal liver function and fulminant hepatic failure) and gastrointestinal (diarrhea, anorexia, nausea and vomiting) [297,305]. In regard to the use of hydroxychloroquine during COVID-19, studies seem to show a relatively safe profile [299,306]. Its efficacy being still not demonstrated, it was removed from treatment guidelines until further studies [307,308].

Systemic corticosteroids have a broad-spectrum immunosuppressive activity. In the context of exacerbated inflammation as we described in SARS-CoV-2, they may be of benefit. Thus, clinicians tend to use it in most critically ill patients. However, such treatment was already used during SARS and MERS outbreaks, and led to delayed viral clearance and detrimental secondary outcomes, such as a prolonged length of stay in intensive care units or bacterial/fungal infections [309,310]. Similar data have been emphasized for SARS-CoV-2 in moderate disease with no clear benefit of corticosteroids use, more adverse outcomes and delayed viral clearance [311,312,313]. However, benefits for a precise corticosteroids therapy in severe COVID-19 patients either pulse corticosteroids or dexamethasone seems beneficial [314,315,316]. Therefore, such therapy could be of use rather at a late stage of ARDS.

The uncontrolled cytokine release in severe patients has been a major concern of therapeutic trials. Anti-cytokine therapy has been used in this condition (anti-IL-6, anti-TNF-α and anti-IL-1) and numerous clinical trials are still ongoing but a few showed promising results [317,318].

IL-6 is one of the most reported cytokines in COVID-19 pathology, notably in severe patients exhibiting higher serum levels than mild cases. Thus, targeting its signaling is thought to be a therapy of choice but at this time variable results are reported and there is no recommendation to use it in routine owing to limited efficacy evidence [304]. To answer this question a phase II trial has been approved with 330 patients using tocilizumab, an anti-human IL-6 receptor blocking monoclonal antibody [319]. Of note, it has been reported that the tocilizumab effect does not compromise at the same time the antiviral immunity in COVID-19 patients [320].

Granulocyte-macrophage colony-stimulating factor (GM-CSF) expression is also known to be upregulated and participate to hyperinflammation in patients suffering from COVID-19 [146,214,219]. Mavrilimumab is a human monoclonal antibody used in rheumatoid arthritis, which binds GM-CSF receptor α and disrupts its downstream signaling, thus blocking GM-CSF inflammatory activity [321]. This monoclonal antibody showed promising preliminary data, when used in addition to standard management [322]. Patients treated with mavrilimumab presented greater and faster improvement than the control group [322]. However, these data need to be reproduced and confirmed on a larger cohort and multicentric, double-blind, randomized, placebo-controlled studies.

COVID-19 patients exert a delayed IFN type I response leading to an exacerbated secondary cytokine response, responsible for disease severity. Supporting this, type I IFN could be an interesting treatment. Lokugamage et al. showed that in vitro SARS-CoV-2 is more sensitive to type I IFN than SARS-CoV. Regarding these results, they suggest IFN administration as a potential treatment [323,324].

IL-1 is a major actor of uncontrolled cytokine release during severe COVID-19 [318]. Anti-IL-1 therapy by anakinra was shown to be beneficial during severe sepsis [325]. During SARS-CoV-2, anakinra treatment improved the clinical outcome and reduced mortality [304,326].

Complement is the first line of defense during infection. As aforementioned, C5a is responsible for PMN recruitment, and thus probably participates to pulmonary injury during SARS-CoV-2 infection. Eculizumab is a monoclonal antibody that binds C5, hence inhibiting C5a and C5b release, as well as the membrane attack complex formation. This monoclonal antibody is predicted to be a potential therapeutic approach [327], according to what has been explored for MERS-CoV in animal models [157].

Clathrin-mediated endocytosis is a possible SARS-CoV-2 way of cell entry and is regulated by members of the numb-associated kinase (NAK) family [325]. Janus kinase (JAK) inhibitors, notably baracitinib used in rheumatoid arthritis, have been evoked as potential therapeutics during COVID-19 through inhibition of the NAK family [325,328]. Their anti-inflammatory effect may be beneficial in reducing cytokine release in severe patients or detrimental by delaying the interferon response [318,325]. Results of case report and small cohort studies seem encouraging with an improvement of moderate to severe cases and need to be pursued [329,330,331].

### 5.3. Stem Cell-Based Therapy

Mesenchymal stem cells (MSC) are known to have immunoregulatory activities and are for this purpose used as an injected therapy in immune-mediated diseases. Based on these immunoregulatory activities and the supposed pathogenesis of COVID-19, MSC or MSC-secretome injection have been envisaged as treatment by several teams [332,333,334,335,336]. Of note, unlike antibody-based therapy, MSC can target at the same time different cytokines, notably due to their diverse secretome, making these cells a therapy of choice [332]. Thus, it appears that injection of ACE2–MSCs improved the outcomes of seven enrolled COVID-19 patients, mainly through their immunomodulating function [333], leading to inflammation markers reduction [336]. Nonetheless, these experiments were conducted on a very limited number of patients and the statement established should be reconfirmed on a larger cohort.

## Figures and Tables

**Table 1:** Main pathologic findings in the context of SARS-CoV-2 infection reported in the literature. DAD: diffuse alveolar damage and AFOP: acute fibrinous and organizing pneumonia.

Organ	Main Reported Pathologic Findings
Lungs	Edema
	Mononuclear inflammatory cells (lymphocytes, plasmocytes)
	Cellular or proteinaceous exudate
	Type II pneumocyte hyperplasia with cytologic atypia
	Vascular congestion
	Fibrinoid vascular necrosis
	Hyaline membrane formation (hallmark of DAD)
	Fibrin deposition with early organization
	Extensive intra-alveolar “fibrin balls” and organizing pneumonia (AFOP)
	Desquamative pneumocytes
Gastrointestinal tract	Plasma cell and lymphocyte infiltrate
	Edema in the lamina propria
	Partial epithelial degeneration, necrosis, shedding of gastric and intestinal mucosa
Liver	Mild microvesicular steatosis
	Mild lobular and portal activity
	Centrilobular sinusoidal dilatation
Heart	Cardiomyocyte hypertrophy
	Degeneration and necrosis of some cardiomyocytes, mild interstitial hyperemia
	Edema
	Infiltration of a small number of lymphocytes, monocytes and neutrophils
Kidney	Diffuse proximal tubule injuries (loss of brush border, vacuolar degeneration, acute tubular necrosis)
	Lymphocyte infiltration
	Glomerular injuries

**Table 2:** Cytokine profile during diverse infections.

Cytokine (pg/mL)	COVID-19 [220] * [126] ^#^	Dengue Fever [221]	Severe Chikungunya [224]	Viral Sepsis [222]	Leptospirosis [223]	Bacterial Sepsis [222]
IFNγ	9 ^#^	772.4 ± 1762.7	12.6 ± 10.8	9.54 ± 19.16	7.2	16.8 ± 39.61
IL-1	5 (no detectable) *	3.3 ± 2.2	209.6 ± 936.8		9.6	
IL-2	8 ^#^		2.5 ± 0.8	1.46 ± 1.04	4	3.2 ± 6.67
IL-6	25.2 (9.6–54.5) *	24 ± 47	671.9 ± 1261.3	63.23 ± 265	74.7	2533 ± 6559
IL-8	18.4 (11.3–28.4) *	21.1 ± 10.1	734.1 ± 1721.9	30.31 ± 38.01	251.1	1249 ± 6944
IL-10	6.6 (5–11.3) *	32.5 ± 54	90.9 ± 417	14.27 ± 16.75	21	205.11 ± 741.85
TNF-⍺	8.7 (7.1–11.6) *		15.1 ± 107.1		4	
IP-10	>1000 ^#^	56.6 ± 278.5	291.8 ± 460	1944 ± 1295		666.72 ± 766
MCP-1	50 *	389.6 ± 929.3	545.4 ± 418.9	494.31 ± 679		4533 ± 1458
MIG			791.7 ± 434.9	660.71 ± 661		1377 ± 1906

IFNγ: Interferon γ; IL-1: Interleukin 1; IL-2: Interleukin 2; IL-6: Interleukin 6; IL-8: Interleukin 8; IL-10: Interleukin 10; TNF-⍺: Tumor Necrosis Factor ⍺; IP-10: Interferon gamma-induced protein 10; MCP-1: Monocyte Chemoattractant Protein 1; MIG: Monokine Induced by Gamma interferon. * Data coming from [220]. ^#^ Data coming from [126].
